# Adjustments to maintenance therapy and the reasoning behind them among COPD outpatients in Austria: the STEP study

**DOI:** 10.1183/23120541.00615-2023

**Published:** 2024-02-05

**Authors:** Florian Vafai-Tabrizi, Ulrich Schwab, Stephan Brecht, Georg-Christian Funk

**Affiliations:** 1 Karl Landsteiner Institute for Lung Research and Pulmonary Oncology and 2nd Medical Department with Pneumology, Klinik Ottakring, Vienna, Austria; 2 A. Menarini Pharma GmbH, Vienna, Austria

## Abstract

**Background:**

Adjustments to COPD maintenance treatment are based on different guidelines. In Austria, there is a lack of real-world data on treatment adjustments of COPD outpatients and their underlying rationale. The STEP study characterised change patterns of pharmacological maintenance therapy in COPD outpatients in predefined categories of step-up, step-down and switch, the underlying reasons, and predictors in clinical routine in Austria.

**Methods:**

STEP was a single-visit non-interventional study in Austria. 77 pulmonologists based in outpatient clinics documented previous and adapted COPD therapy, reason for change, patient characteristics, COPD phenotype, and lung function. Patients’ COPD symptom burden was assessed by using the COPD Assessment Test (CAT). Predictors for therapy changes were identified.

**Results:**

1137 patients were studied (mean±sd age 67±10 years; 56.9% male; mean forced expiratory volume in 1 s 56.3% predicted; Global Initiative for Chronic Obstructive Lung Disease B and E stages 66% and 19%, respectively; mean CAT score 17.5). Therapy step-up was observed in 59.3%, treatment switch in 21.7% and step-down in 19.0% of patients. Triple therapy comprised the biggest proportion of inhalation treatment (53.3%). Physicians reported lung function, symptom burden and exacerbations as the main reasons for step-up or step-down, whereas switches within the same treatment class were predominantly caused by device issues. Predictors for step-up were comorbid asthma and exacerbations among others.

**Conclusions:**

STEP was the first study to investigate COPD therapy changes in clinical routine in Austria. The most frequent treatment adjustment was step-up, followed by treatment switch and step-down. Symptom burden, stable or improved lung function and inhalation device handling were the most frequently given reasons for adjustments.

## Tweetable abstract

**In STEP, most frequently, step-up treatment adjustments to triple therapy were observed, followed by switch within the treatment class and step-down treatment adjustments. COPD symptom burden and other factors accounted for treatment adjustments.** https://bit.ly/3QA1qjZ

## Introduction

COPD is a chronic and progressive disorder of the lung with a prevalence in Austria estimated at more than 1.2 million patients [1, 2], associated with a significant burden of disease [3]. Treatment goals comprise symptom reduction, avoidance of exacerbations, maintaining quality of life and reducing mortality risk. While there is no causal treatment for COPD, there is evidence of the clinical benefit of inhaled maintenance therapy [4]. Current maintenance treatments are typically focused on long-acting bronchodilators and inhaled corticosteroids (ICS). Over the last years, inhaled treatment combinations have become available as fixed-dose combinations of two or three substance classes provided in different inhalation devices and most are accessible in Austria only after an examination by a pulmonologist. COPD is currently viewed as a chronic condition that should be managed by identifying and modifying individual treatable traits [5, 6].

Treatment adherence and correct inhalation technique are crucial to achieve symptomatic improvement [7, 8]. Disease-specific questionnaires used in clinical routine suggest that a significant proportion of Austrian COPD outpatients still remain symptomatic with a mean St George's Respiratory Questionnaire (COPD-specific version) total score of 43.1 while on treatment [9]. Global Initiative for Chronic Obstructive Lung Disease guidelines [8, 10] recommend a COPD management cycle with regular assessment of health status and treatment goals as well as adequate adjustments of treatments. Accordingly, patients should be checked for symptoms, exacerbations, comorbidities, therapy adherence and inhalation technique. Patients with dyspnoea as the dominant symptom should receive dual bronchodilation with a device that is suitable for them. Patients with exacerbations as the predominant trait are candidates for addition of an ICS, depending on blood eosinophil count. Additional options for patients with frequent exacerbations include roflumilast and macrolide antibiotics [8].

As outlined, COPD guidelines suggest a straightforward course of COPD treatment; however, real-world treatment may require complex adjustment due to the interference of patient, physician and health system factors. For example, in Austria inhaled triple therapy use is known to be widespread with up to 77% of COPD patients receiving ICS, irrespective of exacerbation status and phenotype, even before fixed-dose combination therapies became available [11]. Treatment adjustments and reasons for them were described in a stable phase of COPD in clinical routine, providing insights on related factors such as COPD symptoms and exacerbations [12]. The STEP study was conducted to describe directions and reasons for COPD treatment adjustments in Austria when a change in treatment was considered necessary. STEP focused on COPD outpatients treated by pulmonary specialists. Analyses of clinical patient profiles and of physician-reported reasons for treatment adjustments will help to optimise an individualised and evidence-based treatment selection under real-world conditions, which is difficult to achieve for clinical studies with their carefully selected patients.

## Methods

STEP was a single-visit, nationwide, non-interventional study conducted in Austria in two phases from July to September 2021 (summer) and January to April 2022 (winter). Patient data of both phases were merged into one database with summer or winter as preserved variables for the regression models (described in the Statistical analysis section). In total, 77 outpatient pulmonologists across Austria included patients in this study.

Eligible patients had physician-diagnosed COPD and were included in the study by their treating outpatient pulmonologist when a change in maintenance therapy for COPD was deemed necessary. Informed consent was obtained from the participating patients prior to any study-specific documentation.

The study was approved by the Municipal Ethics Committee of Vienna (EK 21-099-VK) and was sponsored by A. Menarini Pharma GmbH.

The collection of clinical data included: age, gender, smoking status and intensity by pack-years, duration of COPD diagnosis, weight and height to calculate body mass index (BMI), common comorbidities according to a real-life cohort study [13], current and worst documented lung function (forced expiratory volume in 1 s (FEV_1_) percentage predicted and FEV_1_/forced vital capacity), number of exacerbations over the last 12 months (including hospitalisations), COPD symptom severity (COPD Assessment Test (CAT) score), COPD phenotypes proposed by Spanish COPD guidelines [14], COPD treatment changes over the last 3 months, and COPD treatment captured by treatment classes (*e.g.* long-acting muscarinic antagonist (LAMA)+long-acting β_2_-agonist (LABA)) before and after treatment adjustment. Three categories of adjustment to treatment were predefined: 1) step-up: treatment escalation defined as increasing the number of treatment classes and/or the respective dosing; 2) step-down: treatment de-escalation defined as reducing the number of treatment classes and/or the respective dosing (ICS withdrawal with a concomitant change in bronchodilators (*e.g.* ICS+LABA changed to LABA+LAMA) was also considered as step-down); and 3) switch: defined as switch within the same therapeutic class. Adjustments to treatment were profiled by the treating physician according to a predefined list of reasons (supplementary table S3), whereby multiple reasons could be selected. Patients were asked to assess their disease symptom severity according to the CAT [15] during their visit at which the adjustment to treatment was prescribed by their treating physician.

### Statistical analysis

Statistical analyses were carried out using the statistical analysis software packages MedCalc (MedCalc Software, Ostend, Belgium) and MS Excel (Microsoft, Redmond, WA, USA). Categorical data are presented as number (percentage). Metric data are presented as arithmetic mean with standard deviation and median (interquartile range (IQR)). Regression models were developed using multivariable regression with stepwise backward variable elimination, where significant variables were entered sequentially and after entering a variable in the model, it was checked and eliminated from the model if it became non-significant. Subgroups were formed *post hoc* and compared using Chi-squared and t-tests in an exploratory manner. p<0.05 was regarded as significant for all tests. To display treatment changes, a Sankey plot was generated.

## Results

### Patient characteristics

**FIGURE 1:**
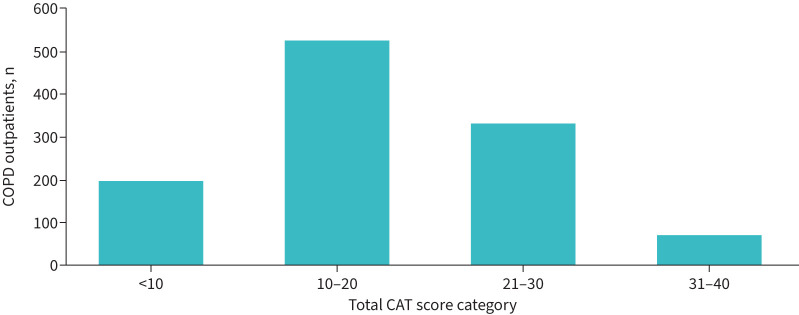
Distribution of COPD Assessment Test (CAT) scores in COPD outpatients at the time of their therapy adaptation (n=1121).

The full analysis set comprised 1137 COPD outpatients with 503 evaluations from the summer phase and 634 evaluations from the winter phase. Patient characteristics are shown in table 1. The majority of patients exhibited pronounced COPD symptoms with CAT scores ≥10 (figure 1).

**TABLE 1:** COPD patient characteristics (n=1137, unless otherwise stated)

**Gender**	
Male	641 (56.9)
Female	485 (43.0)
**Age, years**	66.7±9.9
**BMI, kg·m^−2^**	27±5.5
Summer	503 (44.2)
Winter	634 (55.8)
**Smoking status**	
Current smoker	436 (38.5)
Ex-smoker	614 (54.3)
Never-smoker	81 (7.2)
Pack-years (current and ex-smokers)	40.7±20.0
**Disease duration since COPD diagnosis, years**	
Mean±sd	8.9±6.5
Median (IQR)	8.0 (4–12)
**Comorbidities** **^#^**	
Arterial hypertension	638 (56.1)
Lipid disorder	231 (20.3)
Coronary artery disease	228 (20.1)
Depression	160 (14.1)
Diabetes	158 (13.9)
Asthma	155 (13.6)
Confirmed COVID-19 infection^¶^	118 (10.4)
Obstructive sleep apnoea	99 (8.7)
Heart rhythm disorder	89 (7.8)
Osteoporosis	86 (7.6)
Left cardiac insufficiency	73 (6.4)
Anxiety	57 (5.0)
Active cancer	41 (3.6)
Malnutrition	36 (3.2)
Rhinitis or rhinosinusitis	34 (3.0)
Pneumonia^¶^	34 (3.0)
Stroke in the past	25 (2.2)
Bronchiectasis	25 (2.2)
Other addictive disorders besides smoking	23 (2.0)
Pulmonary hypertension	9 (0.8)
**Lung function: current**	
FEV_1_ % pred (n=1133)	56.3±17.7
FEV_1_/FVC (n=1126)	59.8±14.6
**Lung function: worst**	
FEV_1_ % pred (n=1115)	49.6±16.0
FEV_1_/FVC (n=1105)	56.0±14.2
**COPD exacerbations** **^¶^**	
Any exacerbations	502 (44.7)
Exacerbations	1.6±0.8
Patients with exacerbations requiring hospitalisation	90
Patients requiring oral corticosteroids and/or antibiotics	373
No exacerbations	622 (55.3)
**GOLD COPD stages in patients with FEV_1_/FVC <0.7**	
GOLD A	142 (15.4)
GOLD B	603 (65.5)
GOLD C^+^	12 (1.3)
GOLD D^+^	163 (17.7)
GOLD E^§^	175 (19.0)
**COPD phenotype** **^#^**	
Non-exacerbator	744 (65.4)
Frequent exacerbator	216 (19.0)
Asthma–COPD overlap	155 (13.6)
Patients with emphysema	389 (34.2)
Patients with chronic bronchitis	234 (20.6)
**Change of COPD maintenance therapy (last 3 months)**	
Yes	457 (41.1)
No	656 (58.9)
**CAT score**	
Mean score (n=1121)	17.5±7.9
Step-up	19.0±7.4
Step-down	12.4±6.9
Switch within class	18.1±8.1

Data are presented as n (%), mean±sd or n, unless otherwise stated. BMI: body mass index; IQR: interquartile range; COVID-19: coronavirus disease 2019; FEV_1_: forced expiratory volume in 1 s; FVC: forced vital capacity; GOLD: Global Initiative for Chronic Obstructive Lung Disease; CAT: COPD Assessment Test. ^#^: multiple counting; ^¶^: last 12 months; ^+^: according to GOLD 2022 [10]; ^§^: according to GOLD 2023 [8].

### Maintenance treatment before and after change

**FIGURE 2:**
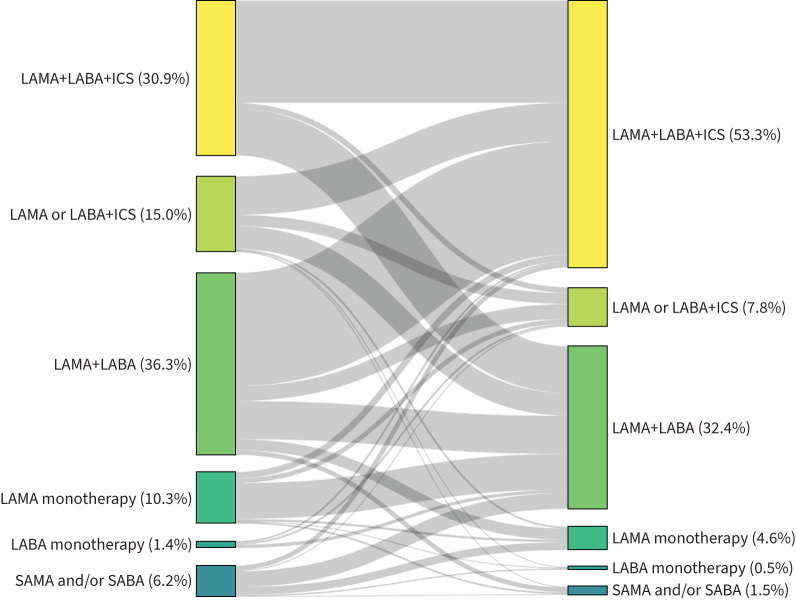
Treatment classes before (left) and after (right) treatment adjustment (n=1081). LAMA: long-acting muscarinic antagonist; LABA: long-acting β_2_-agonist; ICS: inhaled corticosteroid; SAMA: short-acting muscarinic antagonist; SABA: short-acting β_2_-agonist. “SAMA and/or SABA” indicates patients who had SAMA and/or SABA as their sole treatment.

The most prevalent treatment combinations prior to adjustment were LAMA+LABA and triple therapy with 36.3% and 30.2%, respectively. Of 1137 eligible patients, a majority of 59.3% had a step-up in treatment. Step-down and treatment switch within the same class were reported in 19.0% and 21.7%, respectively. Treatment before and after treatment adaptation at the physicians’ discretion is summarised in figure 2 following treatment class listings. The main types of step-up and step-down are shown with their respective frequencies in table 2. Prevalence of ICS-containing therapy was 525 (46.2%) before and 690 (60.7%) after treatment adaptation. Other treatments recorded (as number of patients before/after treatment adjustments) were roflumilast (19/38), theophylline (28/21), macrolide antibiotics (0/4) and mucolytics (49/57). Thus, non-inhaled treatment options such as macrolide antibiotics or phosphodiesterase-4 inhibitors played almost no role in outpatient care (supplementary tables S1 and S2).

**TABLE 2:** Major step-up, step-down and switch therapy changes

**Type of change**	**Before adjustment**		**After adjustment**	**Changes**
**Step-up**	LAMA+LABA	→	LAMA+LABA+ICS	244 (36.3)
	LAMA	→	LAMA+LABA	77 (11.4)
	LAMA/LABA+ICS	→	LAMA+LABA+ICS	82 (12.2)
**Step-down**	LAMA+LABA+ICS	→	LAMA+LABA	98 (45.6)
	LAMA/LABA+ICS	→	LAMA+LABA	35 (16.3)
	LAMA+LABA	→	LAMA	24 (11.2)
**Switch within class**	LAMA+LABA+ICS	→	LAMA+LABA+ICS	159 (64.6)
	LAMA+LABA	→	LAMA+LABA	62 (25.2)
	LAMA/LABA+ICS	→	LAMA/LABA+ICS	13 (5.3)

Data are presented as n (%). LAMA: long-acting muscarinic antagonist; LABA: long-acting β_2_-agonist; ICS: inhaled corticosteroid. “/” indicates “or”.

### Physician-reported reasons and predictors for changes in therapy

The most frequent reasons (multiple nominations were allowed) for step-up of maintenance therapy were ongoing symptoms, limitations of daily activities and declining lung function. In patients with “ongoing symptoms”, additional reasons reported were “limitations of daily activities” (35%), “deterioration of lung function” (20%) and “exacerbations” (19%).

The main reasons for step-down were stable or improved lung function, absence of or seldom exacerbations and scarcity of symptoms. In patients with “stable or improved lung function”, additional reasons reported were “hardly any symptoms” (26%), “absence of or seldom exacerbations” (25%) and “hardly any limitations of daily activities” (23%).

The main reasons for treatment switches were problems with device handling, simplification of therapy and low therapy adherence.

The reasons “blood eosinophil count” and “adverse events” were not rated in any of the three categories as reasons for adjustment of treatment in ≥10% of patients concerned. Common reasons for treatment changes (*i.e.* >10% of patients concerned) as reported by the treating physicians are specified in table 3.

**TABLE 3:** All COPD treatment adaptations and most frequent (*i.e.* >10% of patients concerned) reasons for therapy adaptations

**Treatment step-up: 673 (59.3)**	**Treatment step-down: 215 (19.0)**	**Treatment switch: 246 (21.7)**
Ongoing symptoms: 469 (69.7)	Stable or improved lung function: 155 (72.1)	Handling problems with the inhaler: 105 (42.7)
Limitations in daily life: 351 (52.2)	No or rare exacerbations: 122 (56.7)	Reduction in number of inhalers: 84 (34.1)
Lung function decrease: 260 (38.6)	Almost no symptoms: 115 (53.5)	Suboptimal therapy adherence: 57 (23.2)
Exacerbations: 233 (34.6)	Almost no limitations in daily life: 100 (46.5)	Lung function not matching type of inhalator: 51 (20.7)
Frequent use of bronchodilators: 153 (22.7)	No indication for ICS therapy: 76 (35.3)	Unfavourable dosing scheme: 50 (20.3)
Patient wishes a more intensive therapy: 145 (21.5)	Targeted reduction of ICS use: 61 (28.4)	Others (not further specified): 36 (14.6)
Current exacerbations: 105 (15.6)	Patient wish: 34 (15.8)	

Data are presented as n (%).

In multivariable regression models, predictors for each of the treatment adjustment categories were identified. Significant predictors for any step-up were comorbid asthma, presence of exacerbations, smoking status and comorbidities. In case of step-down, significant predictors for any step-down included absence of comorbid asthma, absence of exacerbations, a lower CAT score and a higher FEV_1_ % pred. Significant predictors for a therapy switch included low FEV_1_, presence of dual bronchodilation and triple therapy (table 4). In contrast, significant seasonal effects were not observed in any of the three subgroups.

**TABLE 4:** Multivariable regression model with stepwise backward variable elimination to identify predictors for changes in maintenance therapy

	**OR (95% CI)**	**p-value**
**Step-up**		
Smoker	1.6677 (1.1438–2.4314)	0.0079
CAT score	1.0776 (1.0478–1.1083)	<0.0001
Exacerbations	2.6267 (1.7717–3.8942)	<0.0001
FEV_1_ as predicted	0.9610 (0.9489–0.9732)	<0.0001
LAMA+LABA	0.0626 (0.0306–0.1281)	<0.0001
LAMA/LABA+ICS	0.0287 (0.0133–0.0621)	<0.0001
LAMA+LABA+ICS	0.0013 (0.0005–0.0031)	<0.0001
Comorbidities	1.1924 (1.0461–1.3592)	0.0084
Asthma	3.0236 (1.7404–5.2531)	0.0001
**Step-down**		
CAT score	0.9058 (0.8772–0.9354)	<0.0001
Exacerbations	0.3179 (0.1985–0.5093)	<0.0001
FEV_1_ as predicted	1.0695 (1.0534–1.0858)	<0.0001
LAMA+LABA	10.3703 (4.1425–25.9609)	<0.0001
LAMA/LABA+ICS	72.4608 (27.4014–191.6167)	<0.0001
LAMA+LABA+ICS	171.9185 (62.8764–470.0647)	<0.0001
Asthma	0.1303 (0.0627–0.2708)	<0.0001
**Switch within class**		
FEV_1_ as predicted	0.9884 (0.9787–0.9982)	0.0203
LAMA+LABA	3.3093 (1.8540–5.9070)	0.0001
LAMA+LABA+ICS	15.4004 (8.8258–26.8724)	<0.0001
**Step-up to triple**		
Exacerbations	2.895 (1.8811–4.4554)	<0.0001
FEV_1_ as predicted	0.9563 (0.9434–0.9694)	<0.0001
SAMA and/or SABA	0.5744 (0.3656–0.9026)	0.0162
LAMA/LABA monotherapy	0.228 (0.1151–0.4519)	<0.0001
LAMA+LABA	3.8128 (2.2011–6.6047)	<0.0001
LAMA/LABA+ICS	2.5342 (1.2838–5.0024)	0.0074
Asthma	2.1375 (1.1367–4.0195)	0.0184

CAT: COPD Assessment Test; FEV_1_: forced expiratory volume in 1 s; LAMA: long-acting muscarinic antagonist; LABA: long-acting β_2_-agonist; ICS: inhaled corticosteroid; SAMA: short-acting muscarinic antagonist; SABA: short-acting β_2_-agonist.

To refine the multivariable model for clinically frequent therapy adaptation schemes, predictors were identified for any therapy step-up to triple therapy (LAMA+LABA+ICS in free or fixed combination; n=609). Significant predictors for step-up to triple therapy included presence of exacerbations, lower FEV_1_ and previous therapy with LAMA+LABA, LABA+ICS or LAMA+ICS. Previous treatment with triple therapy was defined as step-up, when patients experienced either dose increase and/or addition of a substance class (roflumilast, theophylline, macrolide antibiotics or mucolytics).

### Triple therapy subgroup analysis

To characterise the patient population receiving triple therapy after treatment adjustment, this subgroup was compared with patients receiving LAMA+LABA after treatment adjustment. Patients with triple therapy were older (67.6 *versus* 66.3 years), had smoked more (40 *versus* 35 pack-years), suffered more frequently from asthma (18.3% *versus* 4.8%), arrhythmic heart disease (10% *versus* 5.1%) and pneumonia (4.4% *versus* 0.8%), graded poorer regarding current and worst lung function, had more exacerbations, and achieved a higher CAT score (20.3 *versus* 14.1). Regarding the phenotype, patients with triple therapy were more frequently classified as frequent exacerbators (30.8% *versus* 4.8%), and exhibited asthma–COPD overlap (19% *versus* 5.9%), emphysema (40.3% *versus* 28.8%) and chronic bronchitis (23.9% *versus* 17.6%). All comparisons are shown in supplementary table S4.

Furthermore, a multivariable model was defined to identify predictors for ICS withdrawal (table 5). Absence of asthma and no or few exacerbations were identified as the strongest predictors for a change to an ICS-free treatment strategy.

**TABLE 5:** Multivariable analysis identifying predictors for therapy step-down from inhaled corticosteroid (ICS) treatment to no ICS treatment after adaptation (n=203)

	**Coefficient±se**	**Wald**	**p-value**
**Asthma**	−2.21378±0.42994	26.5127	<0.0001
**CAT score**	−0.098339±0.019139	26.4002	<0.0001
**Exacerbations**	−1.12692±0.28215	15.9527	0.0001
**FEV_1_ as predicted**	0.053858±0.63333	6.8237	<0.0001

CAT: COPD Assessment Test; FEV_1_: forced expiratory volume in 1 s.

## Discussion

STEP was the first study to investigate changes in maintenance therapy in COPD outpatients throughout Austria. Most patients had a therapy step-up, of which more than half received triple therapy after the change. However, step-down, including ICS withdrawal, was also a common pathway, as were device changes within the therapeutic class. Common physician-reported reasons for therapy adjustment were COPD symptoms, pulmonary function tests and exacerbations, confirmed by objective predictors for treatment adaptations. Demographic characteristics of STEP patients such as age, gender and BMI are comparable to other COPD real-world studies in Austria [9] as well as to the international, controlled IMPACT study [16], whereas the current mean lung function FEV_1_ (% pred at the last assessment) of 56.3% ranged above the IMPACT study (mean post-bronchodilator FEV_1_ of 45.5%) or a real-world quality of life assessment in Austria with a mean FEV_1_ of 51.5% [9].

In STEP, the high percentage (38.5%) of current smokers was a predictor for step-up. Active smoking is contributing to lung damage and COPD deterioration, which predict the need for additional treatment (step-up). In addition, other Austrian COPD cohorts report a proportion of active smokers >30% [7, 9], leaving room for efforts in smoking cessation programmes. Time since diagnosis was, on average, 8.9 years in STEP, *i.e.* considerably shorter compared with 12 years in the longitudinal German COSYCONET study [17]. Furthermore, CAT scores were very close to other real-world studies conducted in Austria (CAT score at baseline 17.9 [18]) or Germany (CAT score at baseline 18 [17]).

In STEP, the most frequent maintenance treatment after therapy adjustment was triple therapy at 53.3%, whereby ongoing exacerbations were only one reason among others for this treatment choice. Also in Central and Eastern Europe, triple therapy was used in 36.8% in non-exacerbators besides the use in patients with exacerbations and chronic bronchitis [19]. Of note, historical analyses of prescription pathways in the UK between 2002 and 2010 also documented a strong drift towards triple therapy with finally 32% of all COPD patients receiving triple therapy [20]. While this study describes the longitudinal treatment path towards triple therapy, the current STEP study adds physicians' reasons and related patient CAT scoring to treatment adjustment with high triple therapy percentage.

A clinical audit conducted in Spain also identified exacerbations as predictors of an increased probability of step-up and ICS+LABA treatment as an indicator for reduced step-up probability [12]. This clinical audit also identified step-down as a common treatment change, which was determined by FEV_1_ and antibiotic or ICS treatment. The results of STEP found a similar trend regarding FEV_1_. Furthermore, several ICS combinations predicted step-down as well. It is of note that the Spanish clinical audit was conducted in hospital outpatient respiratory clinics and up to 8 years earlier, when other treatment guidelines were in place.

In STEP, symptom burden and exacerbations but also lung function as well as patient preferences and smoking status were important reasons or predictors for treatment adjustments and reveal the personalised nature of clinical decision making.

Escalation of maintenance therapy was the most frequent change within the STEP population. Within step-up the most common treatment change was introduction of inhaled triple therapy (multiple or single inhaler) followed by dual bronchodilation with LAMA+LABA.

The “Rome proposal” recently proposed a uniform definition of exacerbations to provide clinical parameters and consider relevant comorbidities and differential diagnoses [21]. Thus, the use of ICS therapy in patients with exacerbations might change over the next years. However, in the categories of step-down and switch, unwanted side-effects of any treatment were recorded in <10% as reasons for adjustments.

Step-down was also not uncommon in STEP, accounting for 19% of treatment adjustments. Stable lung function, decrease in exacerbations and decrease in COPD symptoms were the most frequent reasons for step-down, while treatment-related adverse events did not play an important role and comorbid pneumonia within the last 12 months was stated only in 34 cases. Given recent critical opinions on ICS use [22], advantages of step-down from ICS are of particular interest [23]. In STEP, lower CAT score, indicating less COPD symptom burden, and improvement in FEV_1_ were identified as weak but significant predictors for step-down. It is interesting to see that the mean CAT score in the step-down group was 6.6 points lower than in the step-up group, indicating a lower symptom burden rated by the patient.

In the switch category treatment, reasons predominantly referred to the use and number of inhalators, with and without suboptimal therapy adherence. It is now well established that incorrect use of inhalers is common and may critically affect COPD treatment [24]. Given the limited effectiveness of educational interventions and training [25], switching inhalers seems to be chosen frequently.

Eosinophilia was mentioned by physicians as the reason for step-up only in 58 patients. Blood eosinophil count was not captured in STEP, but missing laboratory data at the study visit might explain this finding. Similarly, access to comprehensive assessments of COPD phenotype and comorbidities may require diagnostic tools such as diffusing capacity of the lung for carbon monoxide or fractional exhaled nitric oxide, and might be limited for COPD outpatients during their STEP study visit.

Moreover, Austrian regulations about inhaled drug prescription and financial compensation have resulted in a high ICS+LABA use compared with other European countries [20, 26] and contributed to the specific treatment pattern seen in STEP patients. One example of these regulations is the fact that LAMA+LABA combinations require initial prescription by a pulmonologist according to reimbursement criteria of the Austrian healthcare insurance. The multitude of parameters reported to affect treatment changes for STEP patients demonstrates that Austrian pulmonologists aim at targeted therapy decisions for each individual patient based on different phenotypes and clinical parameters as indicated by the congruence of physician-reported reasons with objective predictors identified by the regression analyses.

In real-world clinical practice, there are naturally differences from the treatment algorithms recommended in the guidelines, partly due to the complexity of individual patients. In this context, the need for a comprehensive pulmonological management is stressed, including rehabilitation. In order to explicitly assess guideline adherence a clinical audit would be necessary. This was not the aim of the current study. The observations that the prevalence of dual bronchodilation and triple therapy is increasing, while the prevalence of ICS+LABA or ICS+LAMA is decreasing, indicate guideline conformance. On the other hand, the high ICS prevalence in STEP suggests potential overtreatment. However, STEP focused on patients with the need for treatment adjustment and thus is not representative of all COPD patients in Austria, but is biased towards patients with high symptom burden and exacerbation risk. In summary, our results do not allow conclusions about potential overtreatment on an individual level. The high ICS prevalence in STEP and other Austrian COPD studies is suggestive of ICS overtreatment on an epidemiological basis.

Another potential explanation for the high ICS prevalence is the fact that 14% of patients had comorbid asthma as reported by the treating physician. In accordance, comorbid asthma was a predictor for triple therapy.

The research questions that STEP wanted to answer were 1) which treatment adjustments for COPD, as diagnosed by the treating pulmonologist, can be observed in Austria in the real-world, 2) what are the reasons and predictors for these adjustments, and 3) what is the profile of the patients concerned. Such data were not available for Austria so far and STEP complements existing data on symptom burden in stable COPD [9]. Therefore, STEP was conducted across Austria with outpatient pulmonologists and low-threshold eligibility criteria across a winter and a summer period to allow screening for seasonal effects. Besides demographic patient data, STEP includes disease information rated by physicians, such as most recent lung function, comorbidities and COPD phenotype, as well as patient-rated information by the CAT. Treatment adjustments in the over 1100 COPD patients were well defined and categorised in the three main clusters of “step-up”, “step-down” and “switch within the same therapeutic class”, allowing meaningful clinical characterisation and following individualised treatment strains.

Some limitations of STEP are derived from the study design. Treatment adjustments in hospitalised COPD patients or patients in pulmonary rehabilitation facilities were not assessed. Such patients’ clinical profiles might differ from COPD outpatients. Furthermore, longitudinal outcome data about the clinical effect of the treatment adjustments were not assessed. In STEP, 41.1% of the patients had adjustments to their treatment in the last 3 months, indicating the need for frequent treatment adaptations in a progressive disease. Now, STEP data can substantiate these treatment changes. The German DACCORD study had found that 77.2% of the patients still received the same class of medication after 1 year of observation [27].

### Conclusions

In STEP, real-world COPD outpatient treatment adjustments for Austria were categorised, reasons were provided and predictors were identified. Most frequently, step-up treatment adjustments to triple therapy were observed, followed by switch within the treatment class and step-down treatment adjustments. Symptom burden and other factors accounted for treatment adjustments of patients. Predictors described other relevant patient characteristics to complete the clinical profile of treatment adjustment categories in COPD management.

## Supplementary material

10.1183/23120541.00615-2023.Supp1 **Please note:** supplementary material is not edited by the Editorial Office, and is uploaded as it has been supplied by the author.
Supplementary material 00615-2023.SUPPLEMENT

## References

[1] Schirnhofer L, Lamprecht B, Vollmer WM, et al. COPD prevalence in Salzburg, Austria: results from the Burden of Obstructive Lung Disease (BOLD) Study. Chest 2007; 131: 29–36. doi:10.1378/chest.06-0365 17218553

[2] Firlei N, Lamprecht B, Schirnhofer L, et al. Die Prävalenz der COPD in Österreich – die erwartete Entwicklung bis 2020. [The prevalence of COPD in Austria – the expected change over the next decade.] Wien Klin Wochenschr 2007; 119: 513–518. doi:10.1007/s00508-007-0867-3 17943402

[3] Stolz D, Mkorombindo T, Schumann DM, et al. Towards the elimination of chronic obstructive pulmonary disease: a *Lancet* Commission. Lancet 2022; 400: 921–972. doi:10.1016/S0140-6736(22)01273-9 36075255 PMC11260396

[4] Vogelmeier C, Buhl R, Burghuber O, et al. Leitlinie zur Diagnostik und Therapie von Patienten mit chronisch obstruktiver Bronchitis und Lungenemphysem (COPD). [Guideline for the Diagnosis and Treatment of COPD Patients – Issued by the German Respiratory Society and the German Atemwegsliga in Cooperation with the Austrian Society of Pneumology.] Pneumologie 2018; 72: 253–308. doi:10.1055/s-0043-125031 29523017

[5] López-Campos JL, Centanni S. Current approaches for phenotyping as a target for precision medicine in COPD management. COPD 2018; 15: 108–117. doi:10.1080/15412555.2018.1443064 29558165

[6] Ulrik CS, Vijverberg S, Hanania NA, et al. Precision medicine and treatable traits in chronic airway diseases – where do we stand? Curr Opin Pulm Med 2020; 26: 33–39. doi:10.1097/MCP.0000000000000639 31644440

[7] Humenberger M, Horner A, Labek A, et al. Adherence to inhaled therapy and its impact on chronic obstructive pulmonary disease (COPD). BMC Pulm Med 2018; 18: 163. doi:10.1186/s12890-018-0724-3 30340565 PMC6194635

[8] Global Initiative for Chronic Obstructive Lung Disease (GOLD). Global Strategy for the Diagnosis, Management and Prevention of COPD. 2023. Available from: https://goldcopd.org/

[9] Horner A, Burghuber OC, Hartl S, et al. Quality of life and limitations in daily life of stable COPD outpatients in a real-world setting in Austria – results from the CLARA project. Int J Chron Obstruct Pulmon Dis 2020; 15: 1655–1663. doi:10.2147/COPD.S252033 32764911 PMC7367938

[10] Global Initiative for Chronic Obstructive Lung Disease (GOLD). Global Strategy for the Diagnosis, Management and Prevention of COPD. 2022. Available from: https://goldcopd.org/

[11] Reiger G, Zwick R, Lamprecht B, et al. Phenotypes of COPD in an Austrian population: national data from the POPE study. Wien Klin Wochenschr 2018; 130: 382–389. doi:10.1007/s00508-018-1347-7 29797071

[12] López-Campos JL, Arranz MA, Acuña CC, et al. Determinants for changing the treatment of COPD: a regression analysis from a clinical audit. Int J Chron Obstruct Pulmon Dis 2016; 11: 1171–1178. doi:10.2147/COPD.S103614 27330285 PMC4898035

[13] Raherison C, Ouaalaya E-H, Bernady A, et al. Comorbidities and COPD severity in a clinic-based cohort. BMC Pulm Med 2018; 18: 117. doi:10.1186/s12890-018-0684-7 30012144 PMC6048834

[14] Working Group of the GesEPOC. Guía de Práctica Clínica para el Diagnóstico y Tratamiento de Pacientes con Enfermedad Pulmonar Obstructiva Crónica (EPOC) – Guía Española de la EPOC (GesEPOC). Versión 2017. [Clinical Practice Guideline for the Diagnosis and Treatment of Patients with Chronic Obstructive Pulmonary disease (COPD) – the Spanish COPD Guideline (GesEPOC). 2017 Version.] Arch Bronconeumol 2017; 53: Suppl. 1, 1–64.

[15] Jones PW, Harding G, Berry P, et al. Development and first validation of the COPD Assessment Test. Eur Respir J 2009; 34: 648–654. doi:10.1183/09031936.00102509 19720809

[16] Lipson DA, Barnhart F, Brealey N, et al. Once-daily single-inhaler triple *versus* dual therapy in patients with COPD. N Engl J Med 2018; 378: 1671–1680. doi:10.1056/NEJMoa1713901 29668352

[17] Fischer C, Jörres RA, Alter P, et al. Basic determinants of disease knowledge in COPD patients: results from COSYCONET. Patient Prefer Adherence 2022; 16: 1759–1770. doi:10.2147/PPA.S367284 35923660 PMC9342657

[18] Miravitlles M, Koblizek V, Esquinas C, et al. Determinants of CAT (COPD Assessment Test) scores in a population of patients with COPD in central and Eastern Europe: the POPE study. Respir Med 2019; 150: 141–148. doi:10.1016/j.rmed.2019.03.007 30961941

[19] Koblizek V, Milenkovic B, Barczyk A, et al. Phenotypes of COPD patients with a smoking history in Central and Eastern Europe: the POPE Study. Eur Respir J 2017; 49: 1601446. doi:10.1183/13993003.01446-2016 28495687 PMC5460642

[20] Brusselle G, Price D, Gruffydd-Jones K, et al. The inevitable drift to triple therapy in COPD: an analysis of prescribing pathways in the UK. Int J Chron Obstruct Pulmon Dis 2015; 10: 2207–2217. doi:10.2147/COPD.S91694 26527869 PMC4621207

[21] Althobiani MA, Shah AJ, Khan B, et al. Clinicians’ and researchers’ perspectives on a new chronic obstructive pulmonary disease exacerbation definition: Rome wasn't built in a day. Am J Respir Crit Care Med 2023; 207: 1095–1097. doi:10.1164/rccm.202210-1949LE 36656550 PMC10112434

[22] Agusti A, Fabbri LM, Singh D, et al. Inhaled corticosteroids in COPD: friend or foe? Eur Respir J 2018; 52: 1801219. doi:10.1183/13993003.01219-2018 30190269

[23] Ferreira AJ, Reis A, Marçal N, et al. COPD: a stepwise or a hit hard approach? Rev Port Pneumol 2016; 22: 214–221. doi:10.1016/j.rppnen.2015.12.012 26935750

[24] Usmani OS, Lavorini F, Marshall J, et al. Critical inhaler errors in asthma and COPD: a systematic review of impact on health outcomes. Respir Res 2018; 19: 10. doi:10.1186/s12931-017-0710-y 29338792 PMC5771074

[25] Klijn SL, Hiligsmann M, Evers SMAA, et al. Effectiveness and success factors of educational inhaler technique interventions in asthma & COPD patients: a systematic review. NPJ Prim Care Respir Med 2017; 27: 24. doi:10.1038/s41533-017-0022-1 28408742 PMC5435089

[26] Quint JK, O'Leary C, Venerus A, et al. Prescribing pathways to triple therapy: a multi-country, retrospective observational study of adult patients with chronic obstructive pulmonary disease. Pulm Ther 2020; 6: 333–350. doi:10.1007/s41030-020-00132-7 33064273 PMC7672143

[27] Buhl R, Criée C-P, Kardos P, et al. A year in the life of German patients with COPD: the DACCORD observational study. Int J Chron Obstruct Pulmon Dis 2016; 11: 1639–1646. doi:10.2147/COPD.S112110 27499620 PMC4959587

