# Supplementary material for: Adjustments to maintenance therapy and the reasoning behind them among COPD outpatients in Austria: the STEP study
Source: ERJ Open Res. 2024 Feb 5;10(1):00615-2023. doi: 10.1183/23120541.00615-2023 (PMC10851946; doi:10.1183/23120541.00615-2023)
Supplement: Supplementary file 1 [file 00615-2023.SUPPLEMENT.pdf]

Supplementary Materials

Supplement Table 1: Non-inhaled therapies before therapy adjustment

Therapy before adjustment	N (%)
Total number of patients	96 (8.4%)
Roflumilast	19 (1.7%)
Theophylline	28 (2.5%)
Macrolide antibiotics	0 (0.0%)
Mucolytics	49 (4.3%)

Supplement Table 2: Non-inhaled therapies after therapy adjustment

Therapy after adjustment	N (%)
Total number of patients	120 (10.6%)
Roflumilast	38 (3.3%)
Theophylline	21 (1.8%)
Macrolide antibiotics	4 (0.4%)
Mucolytics	57 (5.0%)

Supplement Table 3: Comprehensive list of reasons for therapy changes provided to physicians. Selection of multiple reasons was allowed.

STEP UP	STEP DOWN	SWITCH
Ongoing symptoms	Stable or improved Lung function	Lack of adherence to therapy
Limitations of daily activities	Hardly any symptoms	Unfavourable dosing regime
Exacerbations	Hardly any limitations of daily activities	Handling problems with the inhaler
Current exacerbation at today's consultation (antibiotics and/or oral corticosteroids are prescribed)	Absence of or seldom exacerbations	Insufficient lung function for current inhaler

STEP UP	STEP DOWN	SWITCH
Patient stopped therapy independently	Absence of an original indication for ICS therapy	Patient refuses the medication
Disproportionate use of short-acting bronchodilators	Intended reduction of ICS therapy	Reduction in the number of inhalers (e.g., change from free to fixed combination)
Deterioration of lung function	Patient actively asks for therapy reduction	Side effects of current Therapy
Targeted increase in ICS therapy	Eosinophil count in blood	Other reasons (unspecified)
Concomitant disease has worsened or new onset	Suspected or confirmed other cause of dyspnea	
Eosinophil count in the blood	Other reasons (unspecified)	
Allergy season or allergen exposure	Side effects of current therapy	
Patient actively asks for therapy intensification		
Specific COPD phenotype		
Other reasons (unspecified)		

Supplement Table 4: Comparison of patients receiving LAMA / LABA vs. triple therapy after therapy switch. Significances are shown by χ^2 (p) or t (p), respectively

Parameter	Characteristic	Key Figure	LAMA / LABA	Triple	Significance
Gender	Male	N (%)	217 (57.9)	337 (57.1)	0.12 (0.73)
	Female	N (%)	153 (40.8)	249 (42.2)	
Age	[Years]	Mean (± SD)	66.3 (9.9)	67.6 (9.9)	1.33 (0.03)
BMI	[kg/m²]	Mean (± SD)	27 (5.3)	26.8 (6.9)	-0.22 (0.54)
Smoking status	Active smoker	N (%)	149 (39.7)	227 (38.5)	2.40 (0.30)
	Former smokers	N (%)	196 (52.3)	328 (55.6)	
	Never-smokers	N (%)	28 (7.5)	31 (5.3)	
	Pack years (active and former	Median	35 (20-50)	40 (25-50)	2.93 (0.03)

Parameter	Characteristic	Key Figure	LAMA / LABA	Triple	Significance
	smokers)	(1st -3rd Quartile)			
Disease duration	Years since diagnosis	Mean (± SD)<br>Median<br>(1st -3rd Quartile)	8.4 (± 6.7)<br>7 (3-12)	9.6 (± 6.6)<br>9 (4-12.8)	1.12 (0.01)
Comorbidities[1]	Arterial hypertension	N (%)	210 (56.0)	341 (57.8)	0.30 (0.58)
	Lipid disorder	N (%)	79 (21.1)	119 (20.2)	0.11 (0.74)
	Coronary artery disease	N (%)	68 (18.1)	133 (22.5)	2.70 (0.10)
	Depression	N (%)	50 (13.3)	89 (15.1)	0.57 (0.45)
	Diabetes	N (%)	52 (13.9)	90 (15.3)	0.35 (0.55)
	Asthma	N (%)	18 (4.8)	108 (18.3)	36.8<br>(< 0.01)
	Confirmed Covid-19 infection[2]	N (%)	36 (9.6)	62 (10.5)	0.21 (0.65)
	Obstructive sleep apnoea	N (%)	29 (7.7)	61 (10.3)	1.84 (0.18)
	Heart rhythm disorder	N (%)	19 (5.1)	59 (10)	7.50 (0.01)
	Osteoporosis	N (%)	23 (6.1)	50 (8.5)	1.80 (0.18)
	Left cardiac insufficiency	N (%)	19 (5.1)	46 (7.8)	2.72 (0.10)
	Anxiety	N (%)	20 (5.3)	29 (4.9)	0.08 (0.77)
	Active cancer	N (%)	19 (5.1)	16 (2.7)	3.63 (0.06)
	malnutrition	N (%)	12 (3.2)	20 (3.4)	0.03 (0.87)
	Rhinitis or rhinosinusitis	N (%)	10 (2.7)	20 (3.4)	0.40 (0.53)
	Pneumonia[2]	N (%)	3 (0.8)	26 (4.4)	10.22<br>(< 0.01)
	Stroke in the past	N (%)	8 (2.1)	13 (2.2)	0.01 (0.94)

Parameter	Characteristic	Key Figure	LAMA / LABA	Triple	Significance
	Bronchiectasis	N (%)	6 (1.6)	18 (3.1)	1.99 (0.16)
	Other addictive disorders besides smoking	N (%)	10 (2.7)	12 (2)	0.41 (0.52)
	Pulmonary Hypertension	N (%)	2 (0.5)	4 (0.7)	0.08 (0.78)
Lung function - current	FEV_1 as predicted	Median (1st -3rd Quartile)	65 (54-74)	48 (37-59.2)	-13.40 (< 0.01)
	FEV_1/FVC	Median (1st -3rd Quartile)	63 (56-70)	56 (46-65)	-1.77 (0.27)
Lung function - worst	FEV_1 as predicted	Median (1st -3rd Quartile)	56 (45-65)	42 (32-53)	-9.64 (< 0.01)
	FEV_1/FVC	Median (1st -3rd Quartile)	59 (51-67)	52 (41-61)	-6.50 (< 0.01)
COPD exacerbations[2]	Any exacerbations	N (%)	94 (25.1)	361 (61.2)	121.56 0.00
	Number of exacerbations	Median (Min - Max)	0 (0 - 2)	1 (0 - 5)	0.49 (< 0.01)
	Number of patients with exacerbations requiring hospitalisation	N	9 (2.4)	75 (12.7)	30.65 (< 0.01)
	Number of patients requiring oral corticosteroids and/or antibiotics	N	64 (17.1)	280 (47.5)	4.46 (0.04)
	No exacerbations	N (%)	278 (74.1)	223 (37.8)	121.56 0.00
GOLD – COPD category	GOLD A	N (%)	41 (10.9)	24 (4.1)	127.88 (< 0.01)
	GOLD B	N (%)	265 (70.7)	249 (42.2)	
	GOLD C[3]	N (%)	63 (16.8)	247 (41.9)	
	GOLD D[3]	N (%)	5 (1.3)	69 (11.7)	
COPD phenotype[1]	Non-exacerbator	N (%)	308 (82.1)	310 (52.5)	87.09 (< 0.01)

Parameter	Characteristic	Key Figure	LAMA / LABA	Triple	Significance
	Frequent exacerbator	N (%)	18 (4.8)	182 (30.8)	94.58 (< 0.01)
	Asthma COPD overlap	N (%)	22 (5.9)	112 (19)	32.95 (< 0.01)
	Patients with emphysema	N (%)	108 (28.8)	238 (40.3)	13.26 (< 0.01)
	Patients with chronic bronchitis	N (%)	66 (17.6)	141 (23.9)	5.39 (0.02)
Change of COPD maintenance therapy (last 3 months)	yes	N (%)	136 (36.3)	255 (43.2)	4.65 (0.10)
	no	N (%)	230 (61.3)	327 (55.4)	
CAT scoring	Mean scoring	Mean (Min - Max) Median (1st -3rd Quartile)	14.1 (1 - 35)	20.3 (4 - 39)	6.26 (< 0.01)

[1] Multiple counting

[2] Last 12 months

[3] according to GOLD 2022 [10]
